# The challenge of linguistic and cultural diversity: Does length of experience affect South African speech-language therapists’ management of children with language impairment?

**DOI:** 10.4102/sajcd.v62i1.71

**Published:** 2015-02-10

**Authors:** Frenette Southwood, Ondene van Dulm

**Affiliations:** 1 Department of General Linguistics, Stellenbosch University, South Africa; 2 Department of Communication Disorders, Canterbury University, New Zealand

## Abstract

**Background:**

South African speech-language therapists (SLTs) currently do not reflect the country's linguistic and cultural diversity. The question arises as to who might be better equipped currently to provide services to multilingual populations: SLTs with more clinical experience in such contexts, or recently trained SLTs who are themselves linguistically and culturally diverse and whose training programmes deliberately focused on multilingualism and multiculturalism?

**Aims:**

To investigate whether length of clinical experience influenced: number of bilingual children treated, languages spoken by these children, languages in which assessment and remediation can be offered, assessment instrument(s) favoured, and languages in which therapy material is required.

**Method:**

From questionnaires completed by 243 Health Professions Council of South Africa (HPCSA)-registered SLTs who treat children with language problems, two groups were drawn: 71 more experienced (ME) respondents (20+ years of experience) and 79 less experienced (LE) respondents (maximum 5 years of experience).

**Results:**

The groups did not differ significantly with regard to (1) number of children (monolingual or bilingual) with language difficulties seen, (2) number of respondents seeing child clients who have Afrikaans or an African language as home language, (3) number of respondents who can offer intervention in Afrikaans or English and (4) number of respondents who reported needing therapy material in Afrikaans or English. However, significantly more ME than LE respondents reported seeing first language child speakers of English, whereas significantly more LE than ME respondents could provide services, and required therapy material, in African languages.

**Conclusion:**

More LE than ME SLTs could offer remediation in an African language, but there were few other significant differences between the two groups. There is still an absence of appropriate assessment and remediation material for Afrikaans and African languages, but the increased number of African language speakers entering the profession may contribute to better service delivery to the diverse South African population.

## Introduction

South African Speech-Language Therapists (SLTs) working in the context of cultural and linguistic diversity face immense challenges in their efforts to provide equitable services to all of their clients (Barratt, Khoza-Shangase & Msimang, 2012). This is in part because the political policies and practices of the past have affected the profession of Speech-Language Therapy and Audiology in a negative manner. A discriminatory education policy resulted in few South Africans of colour entering tertiary education and obtaining professional qualifications (Evans, 1990), and thus few persons of colour entered the profession. Furthermore, research was (and remains) dominated by white intellectuals. Because so-called ‘black universities’ were originally intended to be training centres rather than research centres, few black researchers emerged (Evans, 1990). Furthermore, the *Group Areas Act* (No 41 of 1950, No 77 of 1957, No 36 of 1966) led to residential separation of different racial groups, thereby preventing sustained and integrated contact amongst them. Economic conditions in South Africa have also led to disparity between different ethnic groups. These factors have contributed to a corps of predominantly white SLTs who do not necessarily have in-depth knowledge of the cultural practices of the majority of the country's residents. There is thus a lack of academics and practicing SLTs of colour who might be in a position to offer solutions to practices that perpetuate inequality in access to linguistically and culturally appropriate services.

In South Africa, the challenges that SLTs face in rendering equitable services to all their clients are inherent in every aspect of the intervention process. In this article, we discuss the findings of a national survey pertaining to the practices of South African SLTs during child language assessment and remediation. The survey took the form of a questionnaire sent to all registered SLTs in South Africa, asking them, amongst others, about the languages spoken by their child clients, the language in which they can offer their services, and the assessment material they use. Based on the answers to our questions, we consider whether there is a relationship between length of work experience and the SLT's ability to deal with a culturally and linguistically diverse clientele.

Poorly developed language skills and language impairment can have adverse effects on academic performance (Conti-Ramsden, Knox, Botting & Simkin, 2002). Children whose language problems have been resolved before they enter school may still have poor phonological processing and literacy skills (Stothard, Snowling, Bishop & Chipchase, 1998). Those children who enter school with unresolved language problems experience significant difficulties in all aspects of spoken and written language. Increasingly, over time, they fall further behind their typically developing peers in vocabulary growth (Stothard *et al*. 1998). This is also the case for children from low socioeconomic backgrounds, which is of particular concern in South Africa. These children form a significant percentage of the school-going population, given that 47% of South Africans are living in poverty (Armstrong, Lekezwa & Siebrits, 2008). Such children generally enter school with less well developed language skills in comparison to their middle-class peers (see Raizada, Richards, Meltzoff & Kuhl, 2008), and they often fall behind as they progress through school grades, with the gap between poor and middle-class children ever widening (Cunningham & Stanovich, 1997). Because of the critical influence of language skills on academic success, the nature of the language assessment and remediation services offered to the child population by South African SLTs warrants investigation.

### The problem

Beginning at the most basic level of SLT service provision, Pascoe and Norman (2011) pointed out that the number of qualified SLTs in South Africa is simply too small to adequately service the population. Furthermore, the existing body of SLTs does not reflect the linguistic and cultural diversity of the population it serves, because most SLTs are mother tongue speakers of English or Afrikaans with little or no knowledge of other official languages and/or of the cultural backgrounds of many of their clients (Barratt *et al*. 2012; Pascoe & Norman, 2011). The Royal College of Speech and Language Therapists (1996) guidelines advised that language assessment be carried out in both (or preferably all) languages spoken by a bilingual client. Given that a diagnosis of language impairment cannot be made by assessing a child in his or her second language only (Jordaan & Yelland, 2003), such mismatch between the languages of South African SLTs and their clientele can negatively influence clinical practice. The shortage of SLTs fluent in and knowledgeable about African languages also has implications for the protection of the language rights of South Africans and for language policy implementation (Barratt *et al*. 2012). This study investigated, amongst other things, whether there is a difference in the extent to which more and less recently trained SLTs can offer language assessment and remediation in their clients’ first languages.

With child language assessment, the first step in the intervention process, South African SLTs face a lack of linguistically and culturally appropriate assessment instruments (Pascoe & Norman, 2011; Penn, 1998). For many languages and language varieties spoken in South Africa, no standardised assessment instruments are available. Most of the available assessment instruments are not locally developed and thus contain language structures, cultural references and visual material inappropriate for the local population. The problem might, in some contexts, be partly solved by using informal and dynamic assessment methods, but this is hindered by (1) a lack of knowledge on typical development in many of the mother tongues spoken by the South African clinical population, and (2) a lack of local norms against which a child's performance can be compared (Pascoe *et al*. 2010; Pascoe & Norman, 2011). Valid and reliable assessment is widely acknowledged to be the cornerstone of effective intervention. As such, this lack of linguistically appropriate and culturally unbiased assessment instruments would leave an SLT with limited indications for therapy, even if she or he were able to deliver SLT services in the client's language.

Ethical principles argue against excluding from treatment a client whose language differs from that of the clinician (Pascoe & Norman, 2011). However, in the case of SLT services, a clinician who is not competent in the language and/or does not have linguistically appropriate resources at hand runs the risk of doing more harm to a client than good, amongst others by increasing the risk of under- or over-diagnosis (Gould, 2008).

### Potential solutions

A number of reports have appeared in the literature on steps taken by South African SLTs to address the challenges presented by language barriers in the clinical context. The first is the translation and adaptation of assessment instruments. Whilst a number of authors reported certain levels of success in test translation and adaptation (e.g., Barratt, 2012; Mosdell, 2010; O'Toole & Hickey, 2013), it is a complex process fraught with problems. Test item translation is generally not straightforward because of syntactic, semantic, pragmatic, and/or phonological differences between the two languages. Direct translation may render either, (1) an item which does not target the same structure or skill as in the original language, or (2) an item which is not of the same level of difficulty as the original item. The intricacies of item translation are also often hidden. For example, a change necessitated by a syntactic difference between the languages concerned may lead to the use of a lexical item which has a higher level of difficulty in some dialects than in others. Logistical problems also interfere with the success of test translation. Firstly, it is a decidedly costly process, amongst other reasons because of the need to standardise the translated instrument on a representative sample of the target population. There is a need to re-standardise untranslated English-medium assessment instruments developed elsewhere when used with speakers of South African English (Wilson & Moodly, 2000), and this is also a case for translated instruments. Secondly, despite mutual intelligibility of, and structural similarity between, certain African languages spoken in South Africa, the translation of an adequate range of assessment instruments into each of these languages will be a lengthy and costly task. This is as a result of the number of different languages spoken in South Africa and therefore the number of different languages for which assessment instruments may be needed.

The use of interpreters during assessment and remediation is a second solution often applied in the South African SLT context. Whereas the use of an interpreter is arguably preferable to having no communication at all between client and SLT, it raises its own set of problems. It can also lead to either under- or over-identification of language impairment if the interpreter is not thoroughly knowledgeable about dialectal differences that impact the targets of the intervention. Furthermore, an interpreter may make errors of interpretation that affect either what is communicated or how it is communicated. The SLT remains unaware of any such error and cannot control for its effects (Barratt *et al*. 2012). A further problem with regard to the use of interpreters is a lack of availability. Penn, Mupawosa, and Stein (2009) reported that recently qualified SLTs from the University of the Witwatersrand had no access to formal interpreting services during their Community Service year, although they did report making frequent use of *ad hoc* interpreters. Such use of untrained interpreters exacerbates the problems already pointed out above.

A third solution may lie in appropriate language training for undergraduate SLT students. A brief examination by the authors of the curricula of South African SLT training programs as specified in their online prospectuses indicates that there is some awareness of the need to equip SLT students with knowledge about linguistic and cultural diversity in clinical practice, and with the skills required to cope with such diversity. For instance, some training programmes include course content on issues around multiculturalism and multilingualism. Some cover these topics in clinical speech pathology modules. SLT students who have (Applied) Linguistics as a subject also study bi-/multilingualism as a psycholinguistic and/or sociolinguistic phenomenon. Furthermore, the Psychology modules offered to SLT students at the various universities appear to deal with themes related to core social issues and challenges facing South African society. Regarding SLT students’ linguistic proficiency, at least four training institutions require those who do not have an African language as mother tongue to take an African language as subject, usually for one year. Some institutions also require Afrikaans as subject for those students who have no, or limited, proficiency in Afrikaans. One institution includes the topic *Xhosa communication competence in the clinical context* in its clinical speech pathology modules. For most of the institutions, the prospectuses do not state that an African language is taught or that explicit instruction is given on multilingualism and multiculturalism. The apparent absence of such modules is not necessarily cause for alarm, because (1) relevant content might be inherent in certain speech-language pathology or therapy modules, and (2) students may have the opportunity to acquire some competence in a language other than their mother tongue and some understanding of other cultures naturally during the course of their four years of study. The limited and non-uniform focus on multilingual abilities may, however, indicate that the mismatch between the language backgrounds of South African SLTs and those of their clients is only just beginning to be addressed.

The above discussion suggests that training institutions are taking steps toward addressing the fact that the vast majority of South African SLTs are white speakers of English and/or Afrikaans with little knowledge of other South African languages and cultures. Note, however, that, at feedback sessions in the middle of their Community Service Year, recently qualified SLTs who had graduated from the University of the Witwatersrand and who had studied an African language as part of their undergraduate training reported that such training had not been ‘*particularly useful in practice*’ (Penn *et al*. 2009). The SLTs stated that formal training in an African language should continue, but that ‘on-site language learning had been effective and quick, especially where there was a predominant language spoken (as in KwaZulu-Natal) or when they had attended extra language classes’ (Penn *et al*. 2009).

## Research question

Globally, SLT service delivery to multilingual and multicultural populations is deemed by SLTs to be less than satisfactory (ASHA, 2011a). As stated above, our training institutions are not yet producing large numbers of multilingual and culturally knowledgeable SLTs, and other solutions to effective service delivery to our multilingual population (e.g. test translation and the use of interpreters) are not satisfactory. In light of these facts, the following research question arose: Are SLTs with more years of clinical service or are more recently trained SLTs better equipped to deal with the multilingual and multicultural South African population? On the one hand, the former may be better equipped because they have had a longer time to gain clinical experience in multilingual and multicultural contexts. On the other hand, the latter may be better equipped because (1) they themselves are possibly more diverse in language and culture and (2) they have benefitted from training programmes that deliberately focus on multilingual and multicultural issues. In this regard, it should be noted that we consulted the current prospectuses of the various training institutions and did not conduct an historical overview of their academic and clinical programmes on offer. These prospectuses relate only to the training of the more recently qualified SLTs; we did not systematically investigate the nature of the multilingual and multicultural matters included in previous curricula.

## Methodology

### Aims and objectives

The aim of this study was to investigate whether length of clinical experience as an SLT had an influence on aspects of child language intervention practices, this field being particularly challenging in the multilingual and multicultural context of South Africa. Specific objectives were to analyse data from a survey to ascertain whether there were differences between more experienced (ME) SLTs and less experienced (LE) SLTs with regard to:

- the number of child clients treated (including bilingual child clients), the primary causes of the children's language problems, and the languages spoken by these clients
- the language(s) in which assessment and remediation can be carried out
- the assessment instrument(s) favoured and the reasons for this
- factors taken into account when selecting an assessment instrument
- the language(s) in which therapy material is required.

### Research design

The study took the form of an exploratory and descriptive questionnaire-based survey. Ethical clearance for conducting the research was granted by the Ethics Committee of the Faculty of Arts and Social Sciences at Stellenbosch University. Respondents who completed and returned the questionnaire indicated thereby their consent for participation in the survey.

### Materials

Data to inform the above research question were collected by means of a questionnaire devised by the authors (see Appendix). The first section of the questionnaire requested respondents to indicate their clinical setting(s), their length of service delivery, and their language skills, particularly the language(s) in which they were able to assess and treat clients. The second section posed questions about respondents’ clientele, particularly on how many children with language problems typically existed on respondents’ caseloads, the main cause(s) of the language problems, the ages and first languages of the children, the proportion of the children who were bilingual, and how many of the bilingual children received remediation in their first language. The third section focused on the assessment and remediation of language problems, specifically the assessment instruments used and respondents’ ratings of these with regard to various factors, the factors considered by respondents when selecting an assessment instrument, and the language(s) in which therapy material is required.

The questionnaire contained two different types of questions. Content questions required written answers, such as the number of years spent working as an SLT in South Africa. Response selection questions offered a number of possible response categories, of which a respondent could tick either one or more than one. For example, when indicating the average number of children with language problems on his or her caseload, a respondent could tick only one of several categories (1–5; 6–10; 11–15; 16–20; 20+), but when indicating causes of the language problems, a respondent could tick one or more categories (e.g. language delay, neurological impairment, insufficient exposure to literacy practices, etc.). For questions with an ‘Other’ option, respondents were requested to supply further written information. In order to increase the validity of the questionnaire, care was taken to phrase questions in a simple yet precise manner, to keep questions strictly related to the topic at hand (Kazi & Khalid, 2012), and to include questions that would allow the researchers to answer the research questions. Completion of the questionnaire took approximately 10 minutes.

### Respondents

Paper copies of the questionnaire were posted at the end of January 2012 to every one of the 1915 SLTs registered with the Health Professions Council of South Africa (HPCSA). Total population sampling thus took place. These SLTs excluded community service graduates. Fifty-eight questionnaires were returned undelivered and 272 completed questionnaires were returned in the stamped self-addressed envelopes provided. This comprises a return rate of 15%, with representation from all nine provinces. We acknowledge a possible sample bias, in the sense that possibly only those therapists interested in the topics covered in the survey completed the survey. Such a bias would have influenced the results and limited the generalizability of the findings.

Of the 272 respondents, 243 were South Africa-based SLTs who had children with language problems on their caseloads. Two groups of participants (those with more and those with less experience) were drawn from these 243 completed questionnaires. To ensure two distinctly different groups, those respondents with 20 or more years of experience (average 28 years 9 months; median 30 years 0 months; SD = 5 years 8 months) and those with five or less years were selected. The more experienced (ME) group comprised 71 respondents and the less experienced (LE) group 79. The two groups differed significantly in the time they had practiced in South Africa (*p =* 0.000) but not in the time they had practiced abroad (*p =* 0.398). Regarding their service-delivery setting, 30 of the 79 respondents (38.0%) in the LE group and 54 of the 71 in the ME group (76.1%) worked in private practice. This difference between proportions of each of the two groups working in private vs. public institutions was significant (*p =* 0.000) and should be borne in mind when interpreting the findings.

### Data analysis

Data were transferred from the paper versions of the selected returned questionnaires to a Microsoft^®^ Office Excel file. Total responses of each of the two groups on various questions were tallied. Comparisons between two binary or categorical variables were made using Pearson Chi Square, where a *p*-value < 0.05 was taken to imply a significant association between the two variables, i.e. that there was a difference in proportions between the way in which the two groups responded to the question.

## Findings and discussion

There were no significant differences between the two groups regarding the number of children SLTs had on their caseloads (Chi square 1.591 (*df* = 4), *p =* .810), or average number of children with language difficulties (Chi square 5.100 (*df* = 4), *p* = .404). There were also no significant differences between the two groups regarding the primary causes of their child clients’ language problems in the given options (viz. language delay, language disorder, incomplete second language acquisition, hearing impairment, neuro-logical impairment, insufficient exposure to literacy pra-ctices, under-stimulation), or any other cause mentioned (see Table 1). The only exception was insufficient exposure to school-type language. More LE than ME respondents indicated this as a primary cause of the language difficulties of their child clients.

**Table 1:** Primary causes of language problems of child clients on South African speech-language therapists caseloads.

Primary cause	% (*N*) of SLTs reporting		Chi square (*df* = 1)	*p*-value
	LE group (*N* = 79)	ME group (*N* = 71)		
Language delay	88.6 (70)	93.0 (66)	.836	.360
Language disorder	55.7 (44)	64.8 (46)	1.288	.256
Neurological impairment	49.37 (39)	47.9 (34)	.303	.856
Hearing impairment	31.7 (25)	28.2 (20)	.215	.643
Incomplete second language acquisition	46.8 (37)	49.3 (35)	.091	.763
Insufficient exposure to literacy practices	35.4 (28)	28.2 (20)	.909	.340
Insufficient exposure to school-type language	34.2 (27)	18.3 (13)	4.814	.028
Autism spectrum disorder	5.1 (4)	4.2 (3)	.059	.808
Under-stimulation	2.5 (2)	0.0 (0)	1.822	.177

LE, less experienced; ME, more experienced; SLT, South African speech-language therapists; *df*, degrees of freedom

Turning to the languages spoken by the child clients with language problems: There were no significant differences between the two groups with regard to the number of SLTs who reported seeing child clients who speak Afrikaans or an African language as home language (see Table 2). Considering individual African languages, significantly more ME than LE respondents reported seeing isiZulu-speaking children, but there were no significant differences between the groups for the other African languages reported (this could be related to the fact that isiZulu is the language with the largest number of home language speakers in South Africa; Statistics South Africa, 2012). Significantly more ME respondents than LE respondents reported seeing child clients who speak English as their home language. Considering bilingual children, the difference between the two groups approached significance, with more LE respondents (47.9% of the 76 respondents who answered the question; median 40%; SD 31.4) than ME respondents (36.8% of the 69 respondents who answered the question; median 30%; SD 27.4) seeing bilingual child clients (Mann-Whitney U = 2133.5; *p* = .05336).

**Table 2:** Languages of child clients on South African speech-language therapists caseloads.

Children	% (*N*) of SLTs reporting		Chi square (*df* = 1)	*p*-value
	LE group (*N* = 79)	ME group (*N* = 71)		
With English as home language	58.2 (46)	81.7 (58)	9.681	.002
With Afrikaans as home language	48.1 (38)	52.1 (37)	.241	.624
With an African language as home language	53.2 (42)	50.7 (36)	.091	.763
isiZulu	5.1 (4)	15.5 (11)	4.520	.034
isiXhosa	6.3 (5)	5.6 (4)	.032	.858
Setswana	3.8 (3)	11.3 (8)	3.071	.080
Sesotho	2.5 (2)	4.2 (3)	.333	.564
Siswati	2.5 (2)	0 (0)	1.822	.177
Sepedi	0 (0)	8.5 (6)	6.954	.008
Tshivenda	1.3 (1)	2.8 (2)	.459	.498
isiNdebele	0 (0)	1.4 (1)	1.120	.290
Unspecified African language	31.7 (25)	23.9 (17)	1.100	.294

LE, less experienced; ME, more experienced; SLT, South African speech-language therapists; *df*, degrees of freedom

There were no statistically significant differences between the two groups of respondents regarding ability to evaluate children with language impairments in Afrikaans or English. There were, however, limited indications that LE respondents are better equipped than ME respondents in evaluating such children in languages other than Afrikaans or English: More LE respondents than ME respondents indicated being able to evaluate in an African language (either with or without the use of interpreters). As far as assessment in specific languages is concerned, only the finding for isiZulu was statistically significant – more LE than ME respondents reported being able to evaluate clients in isiZulu (see Table 3).

**Table 3:** Languages in which assessment can be carried out.

Language	% (*N*) of SLTs reporting		Chi square (*df* = 1)	*p*-value
	LE group (*N* = 79)	ME group (*N* = 71)		
English	97.5 (77)	100 (71)	1.822	.177
Afrikaans	65.8 (52)	67.6 (48)	.053	.817
An African language	30.4 (22)	8.5 (5)	11.239	.001
isiZulu	17.7 (14)	1.4 (1)	11.057	.001
isiXhosa	3.8 (3)	1.4 (1)	.822	.365
Setswana	2.5 (2)	1.4 (1)	.241	.624
Sesotho	2.5 (2)	0 (0)	1.822	.177
Siswati	2.5 (2)	0 (0)	1.822	.177
Sepedi	1.3 (1)	0 (0)	.905	.342
Tshivenda	1.3 (1)	0 (0)	.905	.342
Unspecified African language	2.5 (2)	4.2 (3)	.333	.564

LE, less experienced; ME, more experienced; SLT, South African speech-language therapists; *df*, degrees of freedom

With regard to treatment, similar results were obtained as for assessment. Again, there were no statistically significant differences between the two groups regarding ability to remediate in Afrikaans or English. There were, again, some indications that LE respondents are better equipped than ME respondents in treating children with language impairments in other languages: more LE than ME respondents indicated being able to treat in an African language (either with or without the use of interpreters). As was the case for assessment, the only significant finding was for isiZulu, with more LE than ME respondents being able to offer remediation in isiZulu (see Table 4). The average percentage of bilingual children with language problems receiving remediation in their first language did not differ significantly between the LE respondents (47.8% of the bilingual clients; median 50%; SD 34.0) and the ME respondents (42.2% of the bilingual clients; median 35%; SD 33.9), Mann-Whitney U = 2158.0; *p* = .25261.

**Table 4:** Languages in which remediation can occur.

Language	% (*N*) of SLTs reporting		Chi square (*df* = 1)	*p*-value
	LE group (*N* = 79)	ME group (*N* = 71)
English	100 (79)	98.6 (70)	1.120	.290
Afrikaans	68.4 (54)	69.0 (49)	.007	.931
An African language	27.9 (18)	9.9 (5)	7.759	.005
isiZulu	12.7 (10)	1.4 (1)	6.964	.008
isiXhosa	5.1 (4)	0 (0)	3.693	.055
Siswati	2.5 (2)	0 (0)	1.822	.177
Setswana	1.3 (1)	1.4 (1)	.006	.939
Sesotho	1.3 (1)	1.4 (1)	.006	.939
Sepedi	1.3 (1)	0 (0)	.905	.342
Tshivenda	1.3 (1)	0 (0)	.905	.342
Unspecified African language	1.3 (1)	4.2 (3)	1.262	.261

LE, less experienced; ME, more experienced; SLT, South African speech-language therapists; *df*, degrees of freedom

Table 5 summarises the findings for the LE and ME groups’ choices of assessment instruments. Twenty-six instruments were reportedly used with Afrikaans-speaking children and 49 with English-speaking children, excluding SLTs’ own, informal assessment material. Table 5 includes those instruments mentioned for either Afrikaans-speaking or English-speaking children by at least 5% of either the LE or the ME group. As can be seen from this table, the only significant differences between the two groups were the following: When assessing Afrikaans-speaking as well as English-speaking children, more ME than LE respondents frequently used the *Illinois Test of Psycholinguistic Abilities* (ITPA-3; Hammill, Mather & Roberts, 2001) and the *Peabody Picture Vocabulary Test* (PPVT-4; Dunn & Dunn, 2007) (note that the latest versions of instruments are cited here, but respondents did not always indicate which version they use). When assessing English-speaking children, more ME than LE respondents frequently used the *Test of Language Development* (TOLD-4; Newcomer & Hammill, 2008); the *Renfrew Language Scales* (RLS; Renfrew, 1997) (without specifying whether they use the whole battery or selected subtests) and the *Renfrew Bus Story*; the *Language Assessment, Remediation and Screening Procedure* (LARSP; Crystal, Fletcher & Garman, 1981); the *Test for Reception of Grammar* (TROG-2; Bishop, 2003); and the *British Picture Vocabulary Scale* (BPVS; Dunn, Dunn, Sewell & Styles, 2009). Note that more LE than ME respondents used the *Renfrew Action Picture Test* (Renfrew APT) when assessing English-speaking children. More LE than ME respondents also used an Afrikaans translation of the *Test of Auditory Comprehension of Language* (TACL-3; Carrow-Woolfolk, 1998) when assessing Afrikaans-speaking children. The reverse held true for an Afrikaans translation of the *Preschool Language Scales* (PLS-5; Zimmerman, Steiner & Pond, 2011) and the *Afrikaanse Semantiese Taalevaluasiemedium* (AST, ‘Afrikaans Semantic Language Evaluation Medium’; Pretorius, 1989).

**Table 5:** Assessment instruments used with English- and Afrikaans-speaking children.

Assessment instrument	Used with English-speaking children				Used with Afrikaans-speaking children			
	% (*N*) LE (*N* = 79)	% (*N*) ME (*N* = 71)	Chi square	*p*-value	% (*N*) LE (*N* = 79)	% (*N*) ME (*N* = 71)	Chi square	*p*-value
SLT's own informal assessment material	63.3 (50)	52.1 (37)	1.918	.166	55.7 (44)	47.9% (43)	.914	.339
TACL	62.0 (49)	59.2 (42)	.129	.719	45.6 (36)	25.4% (18)	6.634	.036
PPVT	15.2 (12)	59.2 (42)	31.371	.000	3.8 (3)	21.1% (15)	10.634	.001
CELF	36.7 (29)	40.9 (29)	.270	.604	3.8 (3)	9.9% (7)	2.208	.137
ITPA	20.3 (16)	47.9 (34)	12.850	.000	11.4 (9)	26.8% (19)	5.817	.016
TOLD	11.4 (9)	26.8 (19)	5.817	.016	2.5 (2)	7.0% (5)	1.710	.191
Renfrew APT	35.4 (28)	14.1 (10)	9.018	.003	10.1 (8)	5.6% (4)	1.026	.311
Renfrew (subtest[s] unspecified)	6.3 (5)	16.9 (12)	4.159	.041	1.3 (1)	2.8% (2)	.459	.498
Renfrew Word-finding	3.8 (3)	7.0 (5)	.780	.377	0.0 (0)	1.4 (1)	1.120	.290
Renfrew Bus Story	0.0 (0)	8.5 (6)	6.954	.008	0.0 (0)	1.4 (1)	1.120	.290
LARSP	12.7 (10)	32.4 (23)	8.488	.004	6.3 (5)	12.7 (9)	1.780	.182
Reynell	6.3 (5)	11.3 (8)	1.152	.283	2.5 (2)	8.5 (6)	2.595	.107
EOWPVT	7.6 (6)	16.9 (12)	3.067	.080	3.8 (3)	2.8 (2)	.112	.738
PLS	5.1 (4)	9.9 (7)	1.266	.261	0.0 (0)	5.6 (4)	4.573	.032
TROG	2.5 (2)	12.7 (9)	5.663	.017	1.3 (1)	0.0 (0)	.905	.342
Rosetti	2.5 (2)	5.6 (4)	.937	.333	2.5 (2)	2.8 (2)	.012	.914
BPVS	0.0 (0)	7.0 (5)	5.755	.016	0.0 (0)	0.0 (0)	.000	1.000
AST	–	–	–	–	10.1 (8)	28.2 (20)	8.018	.005

LE, less experienced; ME, more experienced; SLT, South African speech-language therapists; TACL, Test of Auditory Comprehension of Language; PPVT, Peabody Picture Vocabulary Test; CELF, -Clinical Evaluation of Language Fundamentals; ITPA, llinois Test of Psycholinguistic Abilities; TOLD, Test of Language Development; APT, Action picture test; LARSP, Language Assessment, Remediation and Screening Procedure; EOWPVT, Expressive One-Word Picture Vocabulary Tests; PLS, Preschool Language Scales; TROG, Test for Reception of Grammar; BPVS, British Picture Vocabulary Scale; AST, Afrikaanse Semantiese Taalevaluasiemedium; ARW, Afrikaanse Reseptiewe Woordeskattoets

The instruments used by the most respondents were the CELF, TACL, ITPA, Renfrew APT, and PPVT. The reasons why SLTs favoured these, as well as the *Afrikaanse Reseptiewe Woordeskattoets* (ARW, ‘Afrikaans Receptive Vocabulary Test’; Buitendag, 1994) and AST, and the aspects of these instruments preferred by the respondents, are discussed below.

The CELF was found by the vast majority of respondents to be either fair, good or excellent in its efficacy in diagnosis and its ability to provide therapy guidelines – in both cases, by all 27 of the LE respondents who answered this question and by 22 of the 23 ME respondents (95.7%) who answered this question. Furthermore, most respondents regarded the CELF to be a comprehensive assessment instrument (all 27 LE respondents; 17 of the 23 ME respondents [73.9%]).

The TACL received similar ratings: It was judged to be either fair or good in its efficacy in diagnosis (all 41 LE respondents; 18 of the 20 ME respondents [90.0%]) and to have either a fair, good or excellent ability to provide therapy guidelines (40 of the 41 LE respondents [97.6%]; 19 of the 20 ME respondents [95.0%]). Furthermore, respondents generally found the TACL easy to score (28 of the 41 LE respondents [68.3%]; 15 of the 20 ME respondents [75.0%]).

All respondents (9 LE and 15 ME) found the ITPA to be fair, good or excellent regarding its efficacy in diagnosis and its ability to provide therapy guidelines. Amongst the LE respondents, 6 of the 9 (66.7%) judged the ITPA to be quick to administer, whereas only 8 of the 15 ME respondents (53.3%) responded likewise. Regarding ease of scoring, the ITPA also received positive ratings (from 6 of the 9 LE respondents (66.7%) and 11 of the 15 ME respondents (73.3%)). The instrument was judged to be comprehensive by 6 of the 9 LE respondents (66.7%), but by only 4 of the 15 ME respondents (26.7%).

The APT was the mostly frequently used subtest of the Renfrew. This subtest was judged fair, good or excellent with regard to its efficacy in diagnosis by 29 of the 30 LE respondents (96.7%) and by all 7 ME respondents. All respondents (30 LE and 7 ME) judged the ability of the Renfrew APT to provide therapy guidelines as fair, good or excellent. Furthermore, 29 of the 30 LE respondents (96.7%) and all 7 ME respondents judged the Renfrew APT to be quick to administer.

All 7 LE respondents and 23 of the 24 ME respondents (96%) regarded the PPVT to be fair, good or excellent with regard to diagnostic efficacy. It was also judged favourably in its ability to provide therapy guidelines: all 7 of the LE respondents reported it to be fair or good, and 22 of the 24 ME respondents (92%) reported it to be fair, good or excellent. Furthermore, the PPVT was rated quick to administer by 5 of the 7 LE respondents (75%) and by 19 of the 24 ME respondents (80%), and easy to score by 5 of the 7 LE respondents (75%) and by 14 of the 24 ME respondents (60%).

Regarding the Afrikaans-medium instruments, the ARW was judged as having either fair or good diagnostic efficacy (by 11 of the 12 LE respondents (91.7%) and by all 7 ME respondents). The AST was judged as fair, good or excellent (by 5 of the 6 LE respondents (83.3%) and by all 14 ME respondents). Both instruments’ ability to provide therapy guidelines was judged as fair, good or excellent (by 9 of the 11 LE respondents (75.0%) and 6 of the 7 ME respondents (85.7%) for the ARW, and by 5 of the 6 LE respondents (83.3%) and all 14 ME respondents for the AST). Furthermore, many respondents judged the ARW to be easy to score (8 of the 12 LE respondents [66.7%] and 6 of the 7 ME respondents [85.7%]).

Regarding factors taken into account when selecting an assessment instrument, significantly more LE (53.2%; 42 of 79) than ME (36.6%; 26 of 71) respondents considered logistical matters such as purchase price and availability (Chi square 4.130; *p* = .042). There were no statistically significant differences for other considerations listed on the questionnaire. It is notable, however, that 63.3% of LE (50 of 79) and 69.0% of ME (49 of 71) respondents reported considering the underlying linguistic base of the instrument when selecting an assessment instrument (Chi square .546; *p* = .460). In contrast, only 5.1% of LE (4 of 79) and 1.4% (1 of 71) of ME respondents reported considering an instrument's cultural and linguistic appropriateness when used in the South African context (Chi square 1.550; *p* = .213). This suggests a severe lack of attention to these aspects, which may be regarded as particularly important in our multilingual and multicultural society.

Finally, it is clear that although there were no statistically significant differences between the two groups regarding the types of therapy material used (commercially available vs. self-devised; Chi square 1.490 (*df* = 4; *p* = .828), more LE than ME respondents reported being in need of therapy materials in languages other than English and Afrikaans (see Table 6). Also, more than twice as many LE than ME respondents indicated that they require therapy material in an African language. This was especially the case for isiZulu, isiXhosa, and Sesotho. As in the case of the Pascoe *et al*. (2010) survey on SLTs’ practices regarding children with speech difficulties, some of the present respondents commented on the lack of appropriate material for use in the South African context.

**Table 6:** Languages in which therapy material is needed.

Language	% (*N*) of SLTs by which reported		Chi square (*df* = 1)	*p*-value
	LE group (*N* = 79)	ME group (*N* = 71)		
English	69.6 (55)	71.8 (51)	.088	.767
Afrikaans	59.5 (47)	52.1 (37)	.827	.363
An African language	50.6 (40)	22.5 (16)	12.618	.000
isiZulu	20.25 (16)	5.63 (4)	6.916	.009
isiXhosa	12.66 (10)	2.82 (2)	4.921	.027
Setswana	7.59 (6)	1.41 (1)	3.217	.073
Sesotho	6.33 (5)	0.00 (0)	4.649	.031
Siswati	3.80 (3)	0.00 (0)	2.751	.097
Sepedi	2.53 (2)	0.00 (0)	1.822	.177
Tshivenda	2.53 (2)	1.41 (1)	.241	.624
isiNdebele	1.27 (1)	0.00 (0)	.905	.342
Unspecified African language	2.53 (2)	11.27 (8)	4.586	.032

LE, less experienced; ME, more experienced; SLT, South African speech-language therapists; *df*, degrees of freedom

## Summary of the findings

In summary, the more and less experienced groups of SLTs did not differ significantly in the number of children and the number and ages of those with language difficulties on their caseloads. Moreover, the number of SLTs who saw child clients with Afrikaans or an African language as home language did not differ significantly (the single exception here was that significantly more LE than ME SLTs saw first language child speakers of isiZulu). Significantly more ME than LE SLTs reported seeing first language child speakers of English, but there was no significant difference between the numbers of bilingual children on their caseloads. Regarding the languages in which the SLTs could provide their services, there were no significant differences between the groups for assessment and remediation taking place in Afrikaans or English, but more of the LE SLTs could render these services in an African language, and in isiZulu in particular.

The participating SLTs reported making use of a wide range of assessment instruments. Those instruments used significantly more frequently by the ME than by the LE SLTs are the AST, BPVS, ITPA, LARSP, PLS, PPVT, Renfrew (where no subtest was specified), Renfrew Bus Story, TOLD, and TROG. Significantly more LE than ME SLTs routinely use the Renfrew APT and the TACL. Regarding language therapy material, there were no significant differences between the groups in the number of SLTs who reported needing such material in English or Afrikaans. However, significantly more LE than ME SLTs needed material in African languages, particularly in isiXhosa, isiZulu, and Sesotho. Most therapists, regardless of level of experience, were aware of the need to consider the underlying linguistic base of the assessment instruments they consider for use, but few considered the cultural and linguistic appropriateness of these instruments. Thus, with regard to linguistic and cultural awareness during assessment instrument selection, level of experience did not bring about a change. Level of experience, furthermore, influenced few other SLT practices asked about in the present survey.

## Discussion

The research question posed in this study was whether SLTs with more years of clinical service are better equipped than SLTs with fewer years of service to deal with the multilingual and multicultural South African population. The data indicate that more of the less experienced than the more experienced SLTs are able to assess and remediate children in isiZulu (but not in any other African language). This selective increase in ability to assess and remediate in languages other than Afrikaans and English may be as a result of a diversification of the profession regarding SLT ethnicity. The diversification is, however, not yet sufficient to render an SLT corps representative of the linguistic and cultural diversity of the country (Barratt *et al*. 2012; Pascoe & Norman, 2011). The slow rate of diversification could be because of a lack of awareness of the SLT profession. In a study conducted with 651 ethnically diverse male and female learners and students in the United Kingdom, Greenwood, Wright, and Bithell (2006) found that one-third of respondents were totally unaware of speech-language therapy, and minority ethnic respondents were significantly less likely than majority ethnic respondents to know that speech-language therapy is a degree course. ASHA (1997–2014) provided an overview of the challenges pertaining to the recruitment and retention of ethnic minorities in a wide range of professions in the United States of America (USA). There are thus indications that the diversification of the SLT profession is a challenge in countries other than South Africa.

The selective increase in ability of our respondents to assess and remediate in languages other than Afrikaans and English may also indicate (1) that more people who had an African language as additional language at school are entering the profession, or (2) that those SLT training programmes offering an African language as part of its curriculum are successful at rendering linguistically more diverse graduates. This finding could be interpreted as an indication that the less experienced group (those who were trained more recently) are better equipped to deal with multilingual clients, or with clients who do not speak Afrikaans or English. However, such an interpretation does not take into account the possibility that SLTs with more experience may have had more time to acquaint themselves with the range of cultural traditions, customs, values, and beliefs within the multicultural South African population. Such experience-based knowledge may make the latter group of SLTs culturally better equipped, despite being linguistically less so. The SLTs’ relevant cultural knowledge was not investigated in the present study, and so it is not possible to conclude that the less experienced group is necessarily better than the more experienced group at dealing with the multilingual and multicultural South African population. In short, knowing a language sufficiently to assess and remediate in that language speaks of linguistic competence (which is required for accurate diagnosis), but not of cultural competence (which is required for interpreting test behaviour and rendering appropriate therapy services). Cultural competence is given increasing prominence in both research studies (Leadbeater & Litosseliti, 2014; and others) and policies of professional bodies (ASHA, 2004, 2011b; Royal College of Speech and Language Therapists Specific Interest Group in Bilingualism, 2007; and others).

Given the importance of a basic understanding of the theoretical foundations of an assessment instrument, it is encouraging that most respondents, regardless of level of experience, were aware of the need to consider the underlying linguistic base of an assessment instrument. However, the finding that few respondents considered the cultural and linguistic appropriateness of such instruments is disconcerting multilingual and multicultural context. The implications of such findings deserve consideration by those responsible for SLTs’ undergraduate training and in continuing professional development programs.

Turning to assessment and remediation practices: More recently trained than more experienced SLTs could offer their services in an African language, but there were few other significant differences between the two groups. There were some differences in the assessment instruments preferred, but not in the use of informal assessment material. There was widespread use of British and American assessment instruments. This included widespread use of Afrikaans translations of these instruments, despite the problems associated with the use of translated instruments, especially those that have not been renormed. These findings indicate a need for linguistically and culturally appropriate child language assessment instruments in Afrikaans, South African English, and the African languages spoken in South Africa.

Penn (1998, p. 266) reviewed the assessment instruments available in the languages spoken in South Africa. She concluded that ‘development of specialised assessment and intervention protocols for diverse languages and cultures … needs to be expanded’. Penn referred to test translations and adaptations carried out prior to 1998 (chiefly into Afrikaans, but also into isiXhosa and Sepedi). She also referred to test development, notably that of the *Afrikaanse Reseptiewe Woordeskattoets* (Buitendag, 1994), which was developed precisely because of the:

need for the acknowledgement of cultural and linguistic -diversity in South Africa, and the … unavailability of non-biased standardised norm-referenced language tests for the identification of language pathology in children who speak a non-standard language. (Buitendag, 1997, p. vii)

Since the publication of the Penn (1998) survey, these efforts have indeed been expanded by researchers such as Barratt (2012), Mosdell (2010), Pakendorf and Alant (1997), Van Dulm and Southwood (2008), and Southwood and Van Dulm (2012). In addition, research into typically developing language development amongst speakers of languages spoken in South Africa has continued. Such research has been ongoing since at least the 1980s. For example, Kunene (1979) studied Siswati, Connelly (1984) and Demuth (1984) Sesotho, Tsonope (1987) Setswana, and Suzman (1991, 1996) isiZulu. More recently, Pascoe and Smouse (2012) reviewed research on isiXhosa acquisition (by, amongst others, Gxilishe, De Villiers & De Villiers, 2007); Bortz (2012) investigated grammatical aspects of Setswana acquisition; Southwood and Van Dulm (2012) focused on Afrikaans and South African English; Klop, Visser, and Oosthuizen (2012) studied Afrikaans-English bilingual language skills; and Nel (in press) and Potgieter (in press) studied grammatical aspects of Afrikaans-English-isiXhosa trilingual language acquisition. Such developmental data will inform the development of appropriate assessment and remediation material that will reduce the reliance of South African SLTs on instruments developed in other countries for other speaker groups. The existence of a range of linguistically and culturally fair assessment and remediation instruments will assist South African SLTs (regardless of their level of experience) in providing equitable services to their multilingual and multicultural clientele.

### Limitations of the study

This was a small scale study in the sense that the data obtained from only 150 SLTs in South Africa were considered. Those SLTs who did complete the questionnaire might, furthermore, have been biased towards the topic under investigation. These two factors limit the generalizability of the findings. Respondents did not always complete each question, and some questions were answered ambiguously. For instance, more than one response category were selected where only one was possible, e.g., some respondents indicated that they use mostly commercially available assessment instruments as well as mostly self-devised informal assessment instruments. Whereas these matters complicated statistical analyses, they did not deduct from overall conclusions drawn on the basis of the findings. Another major limitation was that respondents were not asked about the training they received on multilingual and multicultural matters whilst they were students (as was done in the survey of Scheffner-Hammer *et al*. 2004). Respondents were also not asked about their level of confidence in treating multilingual children. Pascoe *et al*. (2010) asked this of their respondents to a questionnaire on treating speech difficulties. They found that many of the South African SLTs who rated themselves ‘not very confident’ or ‘not confident at all’ when managing multilingual children had more than 10 years of clinical experience. It would have been of value to ask the present respondents this question regarding their multilingual child clients with language problems.

### Clinical implications

Because of their specific language repertoire (consisting primarily of English only or English and Afrikaans), the majority of South African SLTs are at present still underprepared to provide appropriate language assessment and remediation services to our multilingual population. This lack of SLTs who are proficient in an African language is exacerbated by a lack of culturally and linguistically appropriate assessment instruments for use with our child population. It is furthermore exacerbated by a general absence of contextually relevant language therapy material. SLTs’ responses to the survey discussed here indicated that they are aware of the limitations of their current assessment and remediation practices. Several requests were made by the respondents for the development of appropriate instruments that would improve the quality of child language-related services rendered to our diverse population. Whereas more speakers of African languages (first, second, or additional language speakers) entering the profession will contribute to better service delivery, the absence of appropriate assessment instruments and remediation material will necessarily limit this contribution.

## Conclusion

From the findings of the survey, it appears that the language competencies (as far as delivering services in African languages) of the SLT fraternity in South Africa are changing: More SLTs who entered the field in the past five years are able to assess and remediate children with language impairments in an African language than are SLTs who have been delivering their services for two decades or longer. Pascoe and Smouse (2012) stated that:

clinicians within the SLT profession have an ethical responsibility to effectively assess and manage their clients in the client's first language, even where a language mismatch between client and clinician exists.

Even though more SLTs who can work in African languages are entering the profession now than in earlier years, the findings presented here suggest that the majority of child speakers of African languages will still receive language assessment and remediation in a language other than their first. This will make it difficult to disentangle those speakers of African languages who are typically developing but still in the process of acquiring English (or Afrikaans) from those who have underlying language impairment. This is because such a differential diagnosis requires knowledge of the status of the child's language skills in his or her first language. That said it appears that recruiting first or other language speakers of African languages into SLT training programmes will change levels of service delivery more than will specialised training or maturation as an SLT. Such recruitment and the appropriate training of non-mother-tongue speakers of African languages will increase the linguistic and cultural competencies of SLTs. These SLTs will still need linguistically and culturally appropriate assessment and remediation materials if South African children with language impairments are to receive the contextually relevant evidence-based intervention to which they are entitled. Therefore, the said recruitment and training needs to go hand in hand with research that will provide SLTs with the appropriate means of assessment and remediation.
